# Ophthalmology

**Published:** 1877-06

**Authors:** 


### III. Ophthalmology.

*The Effect of Eserine on Glaucomatous Eyes.* Laqueur. (*Centralbl. f. d. Med. Wissen.* 1876, No. 24.)

The experience that the instillation of atropia sometimes has lit up an attack of glaucoma induced Prof. Laqueur to try whether eserine, the alkaloid of the calabar bean, would prove itself also in glaucoma to be the antagonist of atropia. Applying upon the conjunctiva daily three to four drops of a solution of the sulphate of eserine, he noticed after three or four days a decided decrease of the abnormally high tension of the eyeball in five cases of glaucoma simplex. But the professor is not prepared to warrant the permanency of this gratifying result. The applications could be continued during three weeks, without causing the slightest inconvenience.

*Calabar and its Therapeutic Usefulness.* A. WEBER. (*Gralfe's Arch.* XXII., 4.)

As far back as 1869, W. by a series of very ingenious experiments could assure himself of these facts; (1) that the intra-ocular tension of the vitreous humor of the normal eyeball exceeded that of the aqueous humor; (2) that atropia instilled into a normal eye reduced the tension merely of the vitreous humor, while it increased the tension of the anterior chamber; (3) that calabar acted antagonistically to atropia by increasing the tension of the vitreous space and reducing that of the anterior chamber.

The practical inference Dr. W. drew from the above observations was, that calabar ought to be substituted for atropia in all those affections of the cornea which call for a diminution of the pressure upon the posterior surface of the cornea. W. even ventures the opinion that the routine-like treatment of deep conical ulcerations with atropia has necessitated the tapping of the anterior chamber in a good many cases in which the operation could have been avoided by the use of calabar. The affections for which W. tried the use of calabar with success, are:

1. *Keratocele.* Cases of this kind demonstrated conclusively that calabar diminishes the tension of the aqueous humor. Under its use the thinned portion of the cornea which was bulging out like a small bladder, sunk back to the normal level of the cornea, and under the continued use of calabar, the keratocele did not return, though no pressure-bandage was applied. The corneal ulcer filled up with new tissue and healed with a small, smooth cicatrix.

2. *Conical Cornea.* The effect of the calabar upon the abnormal curvature of the cornea could be plainly noticed, and was proved by the permanent amelioration of the sight.

3. *Old maculæ cornea* which resisted all known remedies have cleared up under the influence of calabar, so that W. is satisfied by his numerous trials that calabar is superior to any other remedy for that purpose. Of course, it must be employed several weeks before an effect can be noticed.

4. *Deep and progressive ulcerations* either in the centre or at the margin of the cornea, as they occur in old and debilitated persons, or in children in connection with blennorrhœal conjunctivitis. In these cases the calabar achieved its most brilliant triumphs; it prevented the perforation of the ulcer; it guarded against hernia of the iris and the subsequent staphylomatous expansion of the cornea; it checked the destructive progress of the ulceration and caused the ulcer to rapidly fill up and cicatrize; and it accomplished all this without the aid of bandages or any other means, except the cauterization of the blennorrhœal conjunctiva. While highly lauding the calabar for its excellent effect upon *deep* ulcers in the cornea, Weber did not forget to state that he could not recommend its use in *superficial* and vascular ulcerations of the cornea. Here the good effect of atropia with a proper bandage remains unquestionable.

5. *Peripheral hernia of the iris;* to reduce which calabar has before been used, but Weber applied it also to guard against an iris hernia after all incisions through the periphery of the cornea.

6. *Glaucoma.* In several forms of this disease, the calabar has, as it seemed, permanently checked the further progress of the glaucomatous process; in some cases its use was followed also by an improved vision.

W. experimented in the most cases with the extract of the calabar bean; but since he could obtain the pure sulphate of eserine, he found the results still more striking and brilliant. The eserine is 10 to 15 times more powerful than the extract; one drop of a one per cent. solution of eserine begins after five minutes to develop its effect upon the ciliary nerves and produces within twenty minutes an extreme contraction of the pupil which lasts ten hours. If one drop of the solution of eserine and one drop of a solution of atropia of equal strength was dropped in the eye, the eserine would cause a contraction of the pupil during the first thirty or forty-five minutes; then its influence was conquered by the action of the atropia which, however, could not produce a complete maximum dilitation of the pupil.

*The use of Eserine.* L. V. Wecker. (*Klin Monatsbl. f. Augenheilk.*, XV., 2.)

The neutral sulphate of eserine forms needles of a yellowish white color. These crystals are very hygroscopic, so much so that in an ill-corked bottle they will melt into a yellowish-brown, resin-like substance. Eserine is soluble in water, making a clear solution with slight yellowish tinge, which gradually (in summer, within twenty-four hours, in the colder season, not till the second or third day) changes to a pink color, and finally assumes a deep red hue. This discoloration is due to a process of oxydation, which weakens the peculiar properties of the eserine. Therefore, the solution loses its power in the same rate as the discoloration proceeds. W. has been employing the eserine since 1875, after the extraction of cataracts without iridectomy. Lately he also used a one per cent. solution every half hour or every hour:

1. In extensive ulcerations of the cornea, with pus in the anterior chamber after the removal of the pus through a puncture of the cornea. He has seen cases in which two-thirds of the anterior chamber were filled with pus; this was removed and eserine was faithfully instilled. The next day not a trace of pus could be discovered in the anterior chamber, while the paracentesis of so large a hypopyon was usually followed by a reproduction of pus where atropia was used.

2. In the progressive ulcers (ulcus serpens) of the cornea. The ulcer is cut across after Saemisch's method, but instead of opening the incision every day afterwards, W. does not touch the cornea at all, but simply relies on the energetic use of eserine. The subsequent opacities are less extensive, allowing even in extreme cases a good chance for restoring the eye-sight by means of an iridectomy.

3. In suppuration of the cornea following the extraction of cataract. As soon as the edges of the wound grow hazy, the aqueous humor turbid and the secretion of the conjunctiva is increased, the wound in its entire extent is re-opened with a fine spatula, in order to draw off all the aqueous humor. Eserine is instilled every hour or thirty minutes, and the eye washed frequently with a warm solution of carbolic acid (one part to thousand parts of water). W. holds the suppuration in these cases to be caused by a septic infection, and therefore treats them antiseptically. He also believes in an antiseptic action of the eserine to which more than to the carbolized lotion he is inclined to attribute the good result of his treatment.

*The Antiseptic Property of Eserine and Atropia.* SCHMIDT—RIMPLER. (*Klin. Monatsbl. f. Augenheilkunde*, XV., 127.)

The vaccination of a rabbit's cornea, with the secretion of a blennorrhœal lachrymal sac is always followed by a destructive suppurative inflammation of the cornea. But this specific keratitis does not come on if the blennorrhœal virus has previously been twenty minutes treated with chlorine water, carbolic or salicylic acid. Such experiments prove conclusively the disinfectant effect of these remedies. Similar experiments made with solutions of eserine and atropia gave a negative result. The blennorrrhœal virus did not lose its specific effect by remaining in these solutions from 20 to 50 minutes.

*Keratitis Produced by the Inoculation of Vaccine Virus.* CRITCHETT. (*Receuil d' Ophthalmologie*, January, 1877.)

Critchett had lately been consulted by a confrere, who three weeks previously while vaccinating a child, received the charge of the lancet into his right eye. Dreading the action of the lymph on the cornea and conjunctiva, he at once washed his eye out, but after twenty-four hours the eye began to inflame and an infiltration appeared in the cornea occupying about two-thirds of the external half of that membrane. The form of the lesion did not leave any doubt but that it was a regular vaccine pustule. After three months all inflammation had disappeared and the opacity of the cornea had decreased in extent; but still Critchett believed an iridectomy was necessary in order to restore a somewhat satisfactory sight.

